# Assessment into the usage of levetiracetam in a canine epilepsy clinic

**DOI:** 10.1186/s12917-015-0340-x

**Published:** 2015-02-07

**Authors:** Rowena MA Packer, George Nye, Sian Elizabeth Porter, Holger A Volk

**Affiliations:** Department of Clinical Science and Services, Royal Veterinary College, Hatfield, AL97TA UK

**Keywords:** Dog, Safety, Seizure, Tolerability, Treatment

## Abstract

**Background:**

Retrospective studies can complement information derived from double-blinded randomized trials. There are multiple retrospective studies reporting good efficacy and tolerability of the anti-epileptic drug levetiracetam (LEV) in human patients with epilepsy; however, reports of LEV's tolerability and efficacy in dogs with epilepsy remain limited. The purpose of this retrospective study was to describe the use of LEV in a canine epilepsy clinic and determine the long-term efficacy and tolerability of LEV in veterinary clinical practice. The electronic database of a UK based referral hospital was searched for LEV usage in dogs with seizures. Information and data necessary for the evaluation were obtained from a combination of electronic and written hospital records, the referring veterinary surgeons’ records and telephone interviews with dog owners. Only dogs that were reportedly diagnosed with idiopathic epilepsy were included in the study.

**Results:**

Fifty-two dogs were included in this retrospective study. Two treatment protocols were recognised; 29 dogs were treated continuously with LEV and 23 dogs received interval or pulse treatment for cluster seizures. LEV treatment resulted in 69% of dogs having a 50% or greater reduction of seizure frequency whilst 15% of all the dogs were completely free from seizures. Seizure frequency reduced significantly in the whole population. No dog was reported to experience life-threatening side effects. Mild side effects were experienced by 46% of dogs and a significantly higher number of these dogs were in the pulse treatment group. The most common side-effects reported were sedation and ataxia.

**Conclusions:**

LEV appears to be effective and well tolerated for reduction of seizures.

## Background

Double-blinded, randomized controlled clinical trials to establish efficacy and safety of novel AEDs are of pivotal importance, but are not without limitations due to their often strict dosing and entry requirements, reducing their applicability to the wider population, e.g. geriatric patients or those with multiple co-occurring conditions. In studies of epilepsy treatment in humans post-marketing studies assessing the clinical use of a drug deemed an important tool, with the most valuable data on efficacy and safety thought to be obtained from prospective and retrospective studies that are monocentric, and gather information on long-term anti-epileptic drug therapy in a single centre only [1,2]. Multiple new anti-epileptic drugs (AED) have been developed in the last two decades in human medicine, which have similar efficacy but are safer and better tolerated than older AEDs [3-5]. One such drug is levetiracetam (LEV), for which there are multiple clinical observational studies reporting good efficacy and tolerability in human patients with epilepsy [1,6-8].

Some of the new AEDs in humans, such as gabapentin, pregabalin, zonisamide and levetiracetam have been trialled in dogs with poorly controlled seizures with variable success [9-15]. LEV, a structurally novel AED, is one of the more promising AEDs for canine epilepsy. LEV seems to act by a unique mechanism; modulation of synaptic release of neurotransmitters by binding to the synaptic vesicle protein 2A (SV2A) [16,17]. In addition to its seizure-suppressing activity, previous experiments in chronic epilepsy models in rodents suggested that LEV might also possess anti-epileptogenic or disease-modifying activity [18-21].

Despite its potential, reports of LEVs tolerability and efficacy in epileptic dogs remain limited. Two recent clinical studies of acute seizures showed that LEV had good seizure suppressing activity in the dog. Dogs receiving LEV 24 h prior to undergoing surgical attenuation of extrahepatic congenital shunts had a significantly decreased risk of postoperative seizures [22]. In another study entailing dogs with cluster seizures or status epilepticus, LEV was superior compared to a placebo group in controlling seizure activity [23]. However, our current knowledge of LEV’s efficacy in chronic canine epilepsy is limited to three studies using LEV in dogs with epilepsy refractory to Phenobarbitone (PB) and/or Potassium bromide (KBr) [12,14,24]. Two of these studies showed a good tolerability of the drug, but its efficacy was not as promising long-term as initially anticipated [12,14]. However, it is known from epidemiological studies in human medicine that only very few patients will respond to a third AED, if they have not responded adequately to two standard AEDs [25]. Despite the aforementioned evidence of LEV’s efficacy and tolerability for acute seizures and chronic epilepsy further studies are needed to evaluate LEV’s long-term efficacy and tolerability in canine epilepsy.

The aim of this retrospective study was to:

(i) Describe the way LEV is used to treat epilepsy in a canine epilepsy clinic in a small animal referral hospital.
(ii) Evaluate the long-term efficacy and tolerability of LEV in these dogs.

## Methods

The study was approved by the Animal Care and Ethics committee of the Royal Veterinary College (RVC 2012/P129). The hospital’s electronic records were searched for the terms ‘levetiracetam’, ‘dog’ and ‘epilepsy’ or ‘seizure’ between February 2006 and February 2012. Information and data necessary for the evaluation were obtained from a combination of electronic and written hospital records, the referring veterinary surgeons records and telephone interview with the dog’s owner. Only dogs which were reported in the records to be diagnosed with idiopathic epilepsy (no remarkable findings on interictal neurological examination, haematology, biochemistry, brain magnetic resonance imaging and cerebrospinal fluid examination) and were administered LEV for ≥3 months were included in the study.

Seizures were classified according to the former guidelines of the International League Against Epilepsy, modified for veterinary patients [26,27]. Cluster seizures were defined as an episode where more than one seizure occurred within a 24 h period. Status epilepticus was defined as seizure activity lasting longer than 5 min without gaining consciousness. A consistent history was collected with the help of a questionnaire [14]. The data collected included: signalment, age of dog at the time of the first seizure, age at death (if appropriate), age at diagnosis, age at start of treatment, age at follow up, weight recorded in the hospital, total number of seizures prior to any treatment with an antiepileptic drug (AED), seizure frequency (mean seizure frequency per month; in the case of cluster seizures each seizure was counted as one event) and seizure days frequency (number of days per month at which the dog had at least one seizure in a 24 hours period) prior to administration of an AED, prior to LEV (Keppra, UCB Pharma) and during LEV treatment, seizure severity and intensity, alterations of behaviour, previous and current medications, side-effects seen with LEV and in particular whether there was an increase in the following variables during LEV treatment; sedation, polyphagia, decreased appetite, polydipsia, polyuria, gastrointestinal signs, ataxia, restlessness, aggression and skin reactions.

### Statistical analysis

Data is presented as median with range and interquartile ranges (IQR). Differences between variables of the two treatment protocols were tested with a Fisher’s exact test for categorical variables and the Mann–Whitney U-test for continuous variables. Within groups, comparisons were performed by McNemar test for categorical data and the Friedman test, followed by the Dunn multiple comparison test for continuous data. Univariate analyses for non-parametric data were used to investigate associations between AED-use prior to LEV and other clinical variables on treatment success (either a >50% reduction in seizure frequency or seizure freedom) using Chi-squared and Mann–Whitney U test for categorical and continuous variables, respectively. All tests were used two-sided with *P* < 0.05 being considered statistically significant.

## Results

The search of the RVC’s electronic database revealed 128 dogs with epilepsy for which LEV was either recommended or prescribed. Sixty-four dogs fulfilled the inclusion criteria of which six owners did not give consent to participate in the study and from a further six dogs follow-up data could not be gathered, leaving a study population of 52 dogs in total.

(i) How was levetiracetam used to treat epilepsy in a canine epilepsy clinic?

### Study population

Breeds represented in the study were Golden retriever (*n* = 7), Border collie (*n* = 5), crossbreed (*n* = 6), German shepherd (*n* = 4), Labrador retriever (*n* = 3), Staffordshire bull terrier (*n* = 3), Yorkshire terrier (*n* = 2), Weimeraner (*n* = 2), German shorthaired pointer (*n* = 2), Doberman pinscher (*n* = 2), Cocker spaniel (*n* = 2), Boxer (*n* = 2), Bichon Frise (*n* = 2), Hungarian Viszla, Beagle, Airedale terrier, Jack Russell terrier, Rottweiler, English springer spaniel, Welsh springer spaniel, Cavalier King Charles spaniel, Curly coated retriever and Irish setter (*n* = 1 each).

### Levetiracetam protocols

Two LEV treatment protocols were recognised, ‘maintenance’ and ‘pulse’. There was a trend for clinicians to use pulse therapy more frequently in the more recent years. Twenty-nine dogs were included in the maintenance group and were treated continuously with LEV. Twenty-three dogs received a pulse treatment protocol for cluster seizures (general protocol: an initial dose of ~60 mg/kg after a seizure occurred or pre-ictal signs were recognised by the owner, followed by ~20 mg/kg every 8 h until seizures did not occur for 48 h).

LEV was prescribed for all dogs to improve seizure control apart from one case for which it was prescribed to shorten the post-ictal phase and in five cases it was used as a result of side-effects attributable to KBr and/or PB use (pancreatitis, marked behaviour change, severe ataxia).

The LEV dose for the maintenance group was 19.5 mg/kg three times daily (9–26.8; IQR 17–22.9 mg/kg), for the pulse treatment group the initial dose was approximately three times the 8 hourly maintenance dose of 22.2 mg/kg (10.6-31.3; IQR 19.9-23.8 mg/kg).

### Differences between levetiracetam maintenance and pulse treatment groups

All the dogs in the pulse treatment group experienced cluster seizures and cluster seizures were reported in 83% (95% CI 67.7-98.4%) of the dogs in the maintenance treatment group (P = 0.04). Twenty-four per cent (95% CI 6.6-41.5%) of dogs in the maintenance and 22% (95% CI 3.4-40.6%) in the pulse treatment group had a status epilepticus prior to LEV treatment (*P >* 0.05).

There was no significant difference between the maintenance and the pulse groups for the following variables; weight at start of LEV, age at first seizure, age at follow up, length of epilepsy, total number of seizures prior to treatment, time on PB and KBr prior to LEV treatment and age at start of LEV treatment (Table 1).

**Table 1:** **Signalment of dogs included and relevant history**

**Variable**	**Maintenance (** ***n*** **= 29)**	**Pulse (** ***n*** **= 23)**	**Total (** ***n*** **= 52)**
Sex			
Male	8 (27.4%)	5 (22%)	13 (25%)
Male neutered	9 (31%)	10 (43.3%)	19 (36.5%)
Female	n/a	1 (4.3%)	1 (2%)
Female neutered	12 (41.4%)	7 (30.4%)	19 (36.5%)
Weight at start of LEV (kg)	28.3 (7.5-77.9; IQR 23.3-33.6)	22.8 (3.4-55; IQR 18.6-44.2)	26.3 (3.4-77.9; IQR 19.2-36.2)
Age at first seizure (years)	1.9 (0.2-7.2; IQR 1.4-4)	2.6 (0.8-7.6; IQR 1.5-4.8)	2.6 (0.2-7.6; IQR 1.5-4.1)
Age at follow up/death (years)	6 (1–12; IQR 4.8-8)^a^	6 (2–12; IQR 4.1-7.9)^b^	6.1 (1–12; IQR 4.6-8)
Length of epilepsy (years)	3 (0.1-8.6; IQR 1.5-5.5)	2.3 (0.3-6.9; IQR 1.6-3.2)	3 (0.1-8.6; IQR 1.5-4.9)
Total number of seizures prior AED	2 (0.3-60; IQR 1–3.5)	3 (0.4-20; IQR 2–5)	2.5 (0.3-60; IQR 1.1-5)
Total number of seizures prior LEV	5.3 (0.7-60; IQR 2.5-12)	4 (0.7-15; IQR 2–9)	4.8 (0.7-40; IQR 2–9.8)
Total number of seizures on LEV	2 (0–40; IQR 0.8-5.7)	1 (0–12; IQR 0–4)	1.3 (0–40; IQR 0.5-4.8)
Total number of seizure days prior AED	1 (0.3-7; IQR 1–2.5)	2 (0.3-6; IQR 1.3-3)	1.6 (0.3-7; IQR 1–3)
Total number of seizure days prior LEV	2 (0.7-12; IQR 1.2-4)	1.5 (0.5-5.3; IQR 1–2)	2 (0.5-10; IQR 1–3)
Total number of seizures days on LEV	2 (0–12; IQR 0.6-3)	1 (0–4.7; IQR 0–2)	1 (0–10; IQR 0.4-2.8)
Treatment prior LEV			
No AED	3 (10%)	2 (9%)	5 (10%)
1 AED	8 (28%)	3 (13%)	11 (21%)
2 AED	18 (62%)	17 (74%)	35 (67%)
3 AED	n/a	1 (4%)	1 (2%)
Time on PB prior LEV (days)	336 (2–1724; IQR 101–713)^c^	386 (3–2023; IQR 218–1044) ^d^	343(2–2023; IQR 120–782)
Time on KBr prior LEV (days)	1047 (190–2443; IQR 713–1594)^e^	1073 (330–2999; IQR 660–2449)^f^	1101 (190–2999; IQR 689–1978)
Age at start of LEV (years)	3.8 (1–8.2; IQR 2.4-6)	4.8 (1.5-10.7; IQR 2.6-7.7)	4.2 (1–10.7; IQR 2.5-6.6)
Length of LEV treatment (years)	1.4 (0.3-7.5; IQR 0.8-3.6)	0.8 (0.3-3.4; IQR 0.5-2)	1.1 (0.3-7.5; IQR 0.6-2.3)

^a^Death at follow up (*n* = 15); ^b^Death at follow up (*n* = 8); Not treated with PB when on LEV(^c^ *n* = 3 or ^d^ *n* = 3); Not treated with KBr when on LEV (^e^ *n* = 11 or ^f^ *n* = 4); IQR, Interquartile range; LEV, levetiracetam; AED, Antiepileptic drugs; PB, phenobarbitone; Kbr, potassium bromide; n/a = not applicable.

Ninety per cent of dogs received treatment with an AED prior to LEV (Table 1), with 89.6% of the maintenance group and 91.3% of the pulse group. In addition to maintenance treatment, 29% of dog owners were provided with rectal diazepam tubes to be used for prolonged seizure activity.

(ii) Long-term efficacy and tolerability of LEV in epileptic dogs

#### Results in seizure frequency and pattern

LEV treatment resulted in 69% (95% CI 56.4-81.6%) of dogs having a 50% or greater reduction of seizure frequency with 15% (95% CI 5.3-24.7%) of dogs being free from seizures with a follow up time of 1.2 (0.3-6.4 years) and of 1.4 years (0.3-6.4 years) respectively. There was no significant difference in the number of responders between the maintenance and the pulse treatment group (≥50% seizure frequency reduction, 66% (95% CI 46.6-85.4%) vs. 74% (95% CI 54.3-93.7%); free of seizures, 7% (95% CI 0–17.4%) vs. 26% (95% CI 6.3-45.7); *P* > 0.05).

Forty-two per cent (95% CI 28.6-55.4%) of dogs had a ≥50% reduction of seizure days frequency with a follow up time of 1.3 years (0.3-6.4 years). There was also no significant difference between the two groups in respect of the number of dogs that had a reduction of seizure days frequency (≥50% seizure days frequency reduction, 44% (95% CI 23.7-64.3%) vs. 52% (95% CI 29.5-74.5%); free of seizures, 7% (95% 0–17.4%) vs. 26% (95% CI 6.3-45.7%); *P* > 0.05).

**Figure 1:**
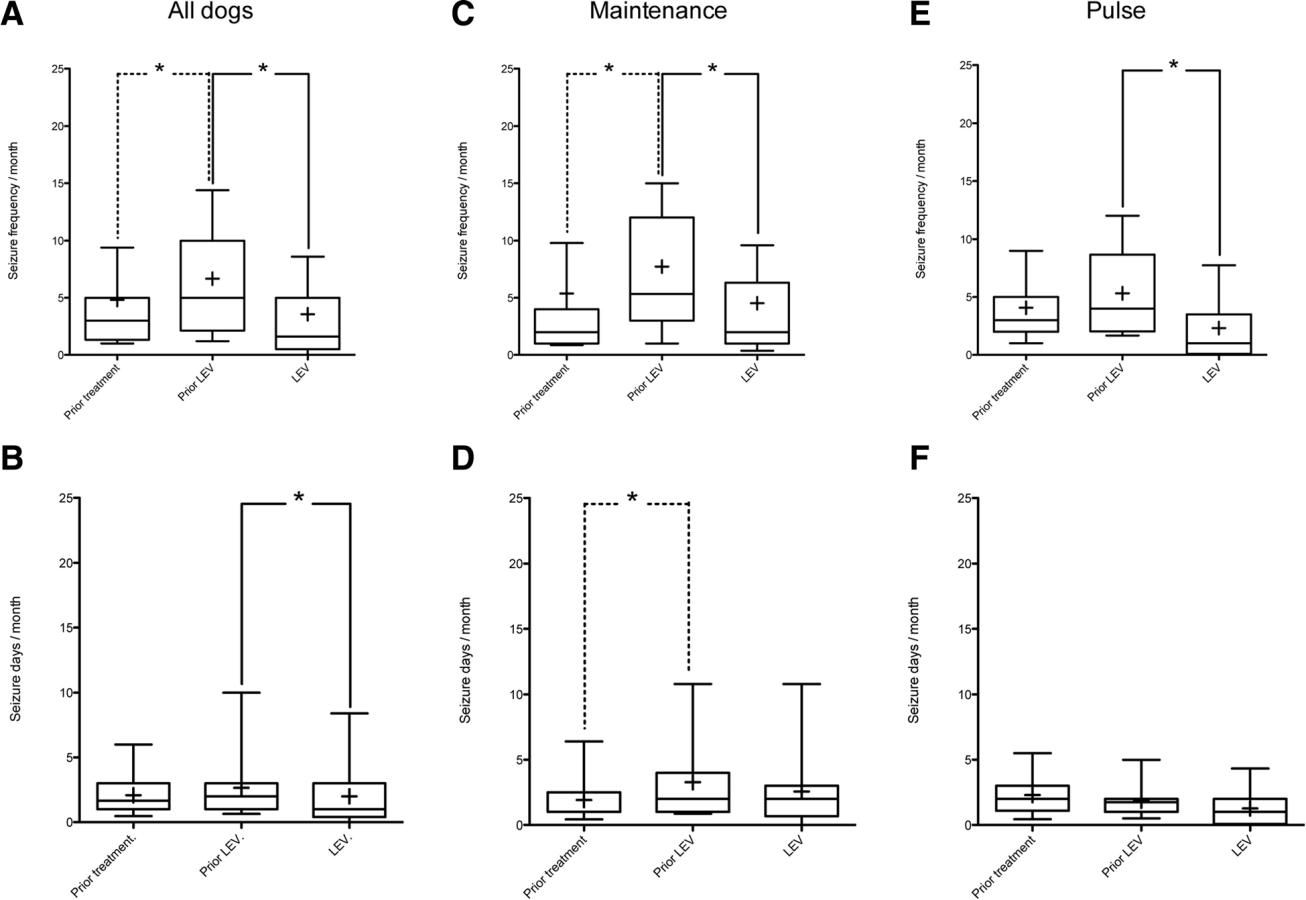
**Seizure frequency and seizure days frequency of all dogs (A, B) and the two treatment groups, maintenance (C, D) and pulse treatment (E, F) group.** The seizure frequency and seizure days frequency are displayed prior to any treatment, prior to levetiracetam treatment (Prior LEV), and while receiving levetiracetam (LEV). Central lines of the box represent the median, lower and upper limits of the box represent the 25th and 75th percentiles and whiskers represent the 10th and 90th percentiles. Mean values are denotated as +. (Friedman test, post-hoc Dunn’s multiple comparison test; *P < 0.05).

The seizure frequency (whole population, maintenance group) and the seizure days frequency (maintenance group) increased prior to LEV treatment compared to before any treatment (Figure 1). LEV reduced the seizure frequency significantly in all groups, but the reduction in seizure days frequency was only significant when analysing the data for the whole population.

The percentage of dogs having cluster seizures increased prior to LEV treatment from 50% (95% CI 36.4-63.6%) to 90% (95% CI 81.9-98.2%) (*P* = 0.02), which was also significant in the pulse treatment group (from 39% (95% CI 17.1-60.9%) to 100%; *P* = 0.02) but not in the maintenance treatment group (from 59% (95% CI 38.9-79.1%) to 83% (95% CI 67.7-98.4%); *P* > 0.05). The number of dogs experiencing cluster seizures decreased on LEV treatment from 90% (95% CI 81.9-98.2%) to 27% (95% CI 14.9-39.1%) (*P* = 0.0001). The dog owners reported that their dog’s seizure severity (maintenance treatment group, 45% (95% CI 24.7-65.3%); pulse treatment group, 43% (95% CI 20.7-65.3%)) and duration (maintenance treatment group, 7% (95% CI 0–17.4%); pulse treatment group, 30% (95% CI 9.4-50.6%) improved during LEV treatment.

### Additional AEDs

Five dogs in the LEV maintenance group did not respond adequately to LEV and zonisamide (*n* = 3) or gabapentin (*n* = 2) was added after 168 days (55–616; IQR 101–539 days). One dog did not respond to LEV in the pulse treatment group and topiramate was added after 92 days.

### Side effects

Life-threatening side effects during the follow-up period was not reported in any of the LEV treated dogs. Forty-six per cent (95% CI 32.5-59.6%) of the dogs in the study experienced side effect during LEV treatment. More dogs in the LEV pulse treatment group were reported to have side effects than in the maintenance treatment group (65% (95% CI 43.6-86.5%) vs. 34% (95% CI 14.6-53.4%), *P* = 0.03). The following side effects were reported to be increased after initiation of LEV treatment; LEV maintenance treatment group: ataxia (n = 5; 17%; 95% CI 1.7-32.4%), polyphagia (n = 3; 10%; 95% CI 0–22.3%), sedation (n = 3; 10%; 95% CI 0–22.3%), polydipsia (n = 1; 3%; 95% CI 0-10%), vomiting (n = 1; 3%; 95% CI 0-10%) and diarrhoea (n = 1; 3%; 95% CI 0-10%); LEV pulse treatment group: Ataxia (n = 10; 43%; 95% CI 20.7-65.3%), sedation (n = 9; 39%; 95% CI 17.1-60.9%), polyphagia (n = 3; 13%; 95% CI 0–28.1%), polydipsia (n = 2; 9%; 95% CI 0–21.9%), aggression (n = 1; 4%; 95% CI 0–12.8%) and restlessness (n = 1; 4%; 95% CI 0–12.8%).

### Mortality

Mortality was not significantly different between the maintenance (52% (95% CI 31.6-72.4%)) and the pulse treatment groups (35% (95% CI 15.5-54.5%)) in the proportion of dogs having been euthanized at follow-up. Seventy-four per cent (95% CI 53.7-94.3%) of these dogs were euthanized because the owners perceived the seizure control not to be sufficient.

### Influence of prior AED treatment

No associations were found between AED-use prior to LEV and treatment success (either a >50% reduction in seizure frequency or seizure freedom), including length of previous epilepsy treatment, time on PB or KBr prior to LEV. There was no difference in seizure frequency or seizure days frequency, or presence of cluster seizures between dogs that achieved a >50% seizure frequency reduction or remission, indicating that pharmacoresponse to LEV is not dependent on seizure type.

## Discussion

The results of this retrospective study provide further evidence that LEV is a well tolerated, seizure-suppressing drug in dogs with epilepsy when used as a maintenance or pulse therapy. Spontaneous and drug-induced epilepsy remission rates in human medicine are around 63% [25], which is markedly higher than most reported in veterinary medicine which range between 14 and 24% [28-31]. Sixty-nine per cent of the dogs had a 50% or greater reduction in seizure frequency including 15% of dogs having no further seizures in the LEV treatment period. Our results are similar to the findings from clinical studies assessing the overall usage of LEV in human neurology practice where the percentage of seizure-free patients ranges from 11-32% [1,6,7]. Using LEV as a monotherapy the seizure-free population was 49% in a recent clinical retrospective study in human medicine [8], which is comparable to the 56% of seizure free patients seen in a randomized controlled trial comparing LEV monotherapy to carbamazepine [32]. However, it needs to be considered that in our study, one of the eight seizure-free dogs on LEV had a follow up of less than 6 months and five dogs of less than 1 year. It is possible that the number of seizure-free dogs might decline should the follow-up period be extended.

Ninety per cent of the dogs had cluster seizures prior to LEV treatment. In a recent epidemiological study, 38% of dogs with epilepsy had cluster seizures [33], however in referral populations the number of dogs presenting with cluster seizures is usually higher. In a separate study 64% of the epileptic dogs presented at a UK referral hospital had cluster seizures [28]. It is generally accepted that cluster seizures are more challenging to control and therefore referral to a specialist hospital could be more likely. The cluster seizure population in this retrospective study is higher than previously reported and it can be assumed that the clinicians in this study used LEV mainly for dogs presenting with cluster seizures. Interestingly, a recent study documented that LEV was superior over placebo in controlling cluster seizures and prolonged seizure activity [23]. Forty-four per cent of dogs in this study were treated with a pulse treatment protocol for cluster seizures. The initial dose of 60 mg/kg was also used successfully in the study from Hardy et al. [26].

The introduction of the pulse treatment protocol was developed after our former study showed that some dogs develop a tolerance to LEV when used chronically [14]. In human medicine, a recent study has shown that LEV had a high efficacy in the first weeks of treatment which was followed by a lower but more stable efficacy in the following weeks [34]. This phenomenon, the ‘honeymoon effect’, has been documented for zonisamide and LEV in dogs with epilepsy [14,15]. Prolonged treatment with LEV has also been shown to induce tolerance in rodent epilepsy models [35]. Pharmacokinetic tolerance is associated with multiple factors provoked by drug administration. These include induction of drug-metabolizing enzymes and increase of multidrug transporters in the liver and kidney. Concurrent PB administration to LEV can increase the clearance of LEV significantly in dogs [36]. Furthermore, pharmacodynamic tolerance is not an uncommon clinical occurrence. Despite no change in drug treatment, seizures can reoccur in human patients following several months of seizure freedom. Factors other than tolerance have to be considered for the return of poor seizure control, such as progression of epilepsy, poor owner compliance, placebo effect of using a novel AED, natural fluctuations of seizure frequency, regression to the mean and acquired drug resistance mediated by mechanisms other than tolerance [35,37]. In our former study [14] we used a previously described strategy to overcome tolerance [35]. During the former study one dog developed tolerance to LEV and the medication was ceased. Following this, only the dog’s cluster seizures were intermittently treated successfully with the aforementioned LEV pulse treatment protocol. The current study supports this clinical approach, as the pulse treatment protocol was as effective in controlling cluster seizures.

Pulse treatment was associated with more side effects post LEV administration than seen in the maintenance group. This could be secondary to the threefold initial LEV dose (60 mg/kg). Another explanation could be that the owners were not able to discriminate between the side effects caused by LEV and the changes seen as a result of seizures. However, overall the drug was well tolerated. No serious life-threatening side effects were reported. The most common side effects associated with LEV in this study were sedation and ataxia which were around three times more common in the pulse treatment group compared to the maintenance treatment group. This is similar to previous clinical and toxicity studies [12,14,23,38]. Long-term toxicity studies revealed that LEV in dogs is extremely safe [38]. Oral LEV can be given in doses up to 1200 mg/kg/day for one year with minor side effects. Some dogs developed gastrointestinal signs. One dog in the current study also had an episode of vomiting and diarrhoea, which the owner associated with the LEV administration. In human medicine, LEV also has been recognised to have a wide margin of safety [39]. Side effects that commonly occur in people within their first month of maintenance LEV treatment are usually not dose-dependent, mainly mild-to-moderate, frequently resolve without cessation of the medication and are transient when the medication is stopped [39,40]. The side effects reported in humans with LEV are similar to those seen in the dog such as sedation, restlessness, gastrointestinal irritations, ataxia, and neuro-behavioural problems in a small percentage [5,39,40]. Neuro-behavioural problems, such as aggression occur mainly in children with a former psychiatric history [39,40]. In a small proportion of dogs in this study the owner reported an increase in restlessness and aggression. This could be associated with LEV, but it can also be secondary to the epilepsy. It was formerly reported that epilepsy can cause abnormal behaviour and that pharmacoresistant dogs are more likely to show signs of aggression [41].

In a former study, eight dog owners documented that their dogs became more lively and interactive on LEV treatment [14]. In the current study this effect on the dogs’ behaviour was not seen. However, a recent study has demonstrated that the owners perceived an improved quality of life when treated with LEV compared to placebo [12].

A main predictor for drug-resistance is a high seizure frequency prior to treatment [25,30,42,43]. In the current study the dogs had a significant increase in seizure frequency from three to five seizures per month prior to LEV treatment. As aforementioned most dogs also had cluster seizures. Despite the high seizure frequency and cluster seizures, 67% of dogs had a decrease in seizure frequency by 50% or more. This is a higher success rate than the results of a recent study, which failed to show a significant change in seizure frequency comparing LEV to a placebo group [12]. The trial was designed as a cross over study, however, as many dogs dropped out of the study the authors could not perform a cross-over comparison and only compared the initial placebo to the LEV group. Interestingly, LEV showed a significant decrease to the baseline seizure frequency, which the placebo group did not. Another former study showed a good initial response, which decreased over time [14]. In the current study, the population was not restricted to dogs with drug-resistant epilepsy to two or more AEDs. The more heterogeneous population in this study could explain the higher long-term success rate.

## Conclusions

There is no doubt that double-blinded, randomized controlled clinical trials are of pivotal importance to establish efficacy and safety of novel AEDs, however, in recent years studies assessing the use of AEDs in practice are increasingly accepted to provide additional useful data in human medicine. The clinical study population is heterogeneous and as the groups are not as standardized as in a prospective blinded trial, which is a significant limitation when drug efficacy is evaluated. However, it is interesting that despite studying a heterogeneous population, potentially useful data was gathered which showed that LEV has a good tolerability and is potentially efficacious in treating epilepsy in the dog. Based on the current study LEV pulse treatment protocol should be considered as an alternative for LEV maintenance therapy. Future studies are needed to confirm this finding.

## Abbreviations

LEV: Levetiracetam
AED: Antiepileptic drug
PB: Phenobarbitone
KBr: Potassium bromide
IQR: Interquartile range
95% CI: 95% Confidence interval
